# Increases in Naloxone Administrations by Emergency Medical Services Providers During the COVID-19 Pandemic: Retrospective Time Series Study

**DOI:** 10.2196/29298

**Published:** 2021-05-27

**Authors:** Dalia Khoury, Alexander Preiss, Paul Geiger, Mohd Anwar, Kevin Paul Conway

**Affiliations:** 1 Research Triangle Institute International Research Triangle Park, NC United States; 2 North Carolina Agricultural and Technical State University Greensboro, NC United States; 3 National Institute of Mental Health Bethesda, MD United States

**Keywords:** opioids, naloxone, EMS, emergency medical services, COVID-19, pandemic, medical services, overdose, outcomes, opioid crisis, public health

## Abstract

**Background:**

The opioid crisis in the United States may be exacerbated by the COVID-19 pandemic. Increases in opioid use, emergency medical services (EMS) runs for opioid-related overdoses, and opioid overdose deaths have been reported. No study has examined changes in multiple naloxone administrations, an indicator of overdose severity, during the COVID-19 pandemic.

**Objective:**

This study examines changes in the occurrence of naloxone administrations and multiple naloxone administrations during EMS runs for opioid-related overdoses during the COVID-19 pandemic in Guilford County, North Carolina (NC).

**Methods:**

Using a period-over-period approach, we compared the occurrence of opioid-related EMS runs, naloxone administrations, and multiple naloxone administrations during the 29-week period before (September 1, 2019, to March 9, 2020) and after NC’s COVID-19 state of emergency declaration (ie, the *COVID-19 period* of March 10 to September 30, 2020). Furthermore, historical data were used to generate a quasi-control distribution of period-over-period changes to compare the occurrence of each outcome during the COVID-19 period to each 29-week period back to January 1, 2014.

**Results:**

All outcomes increased during the COVID-19 period. Compared to the previous 29 weeks, the COVID-19 period experienced increases in the weekly mean number of opioid-related EMS runs (25.6, SD 5.6 vs 18.6, SD 6.6; *P*<.001), naloxone administrations (22.3, SD 6.2 vs 14.1, SD 6.0; *P*<.001), and multiple naloxone administrations (5.0, SD 1.9 vs 2.7, SD 1.9; *P*<.001), corresponding to proportional increases of 37.4%, 57.8%, and 84.8%, respectively. Additionally, the increases during the COVID-19 period were greater than 91% of all historical 29-week periods analyzed.

**Conclusions:**

The occurrence of EMS runs for opioid-related overdoses, naloxone administrations, and multiple naloxone administrations during EMS runs increased during the COVID-19 pandemic in Guilford County, NC. For a host of reasons that need to be explored, the COVID-19 pandemic appears to have exacerbated the opioid crisis.

## Introduction

The United States remains in an unrelenting opioid crisis. According to the latest official mortality data from the Centers for Disease Control and Prevention (CDC), 49,860 Americans died from an opioid-related overdose in 2019 (~137 per day), a substantial increase from 2018 [1]. Furthermore, there is mounting concern that the COVID-19 pandemic will exacerbate the crisis [2,3]. Provisional data indicate that 81,003 people died from a drug overdose during the 12 months ending in May 2020, the highest ever recorded in a 1-year period in the United States and a trend driven largely by heroin and synthetic opioids [4].

The magnitude and volatility of the opioid crisis warrants up-to-date data to monitor and guide intervention in the quickest manner possible, especially during potentially aggravating circumstances such as the COVID-19 pandemic [5]. However, the primary data used to track the opioid crisis in the United States (ie, verified opioid-related deaths from the CDC) are outdated by at least a year, a lag that substantially limits their value for surveillance purposes. Alternative timelier data have been leveraged by several studies to explore the opioid crisis generally [6,7] and how it has shifted since the onset of the COVID-19 pandemic. Niles et al [8] analyzed national urine drug testing patterns before and during the pandemic, and reported a significant increase during the pandemic especially for fentanyl and heroin. Wainwright et al [9] similarly reported a significant increase in positive urine tests during the pandemic for all drugs tested among a convenience sample of patients with or at risk for substance use disorders. Like Niles et al [8], the proportional increase reported by Wainwright et al [9] was greatest for fentanyl and heroin. Using data from the National Emergency Medical Services (EMS) Information System, a national registry of EMS agencies representing 80% of EMS dispatches across 47 states, Friedman et al [10] reported an increase in overdose-related cardiac events from mid-March through August 1, 2020. This surge in cardiac events corresponded temporally with an increase in social distancing as measured by cell phone mobility. Slavova and colleagues [11] examined changes in the number of EMS runs 52 days before and after the declaration of a state of emergency in Kentucky on March 6, 2020, and reported a significant increase in EMS runs in response to opioid overdoses after the declaration. The increase in opioid overdose–related EMS runs during COVID-19 reported by both Friedman et al [10] and Slavova et al [11] is especially noteworthy in the context of an overall decrease in EMS responses across the United States during the early part of the COVID-19 outbreak [12].

In addition to their timeliness, EMS data are also potentially valuable for studying the opioid epidemic because EMS providers increasingly use naloxone, an opioid antagonist medication, to treat respiratory depression in patients with suspected opioid overdose [13-15]. Naloxone administrations have been shown to hold promise as a near real-time proxy of the opioid epidemic [16-20]. Positive associations between EMS naloxone administrations and fatal and nonfatal overdose events have been reported [13,21]. Furthermore, the percentage of patients receiving multiple naloxone administrations has increased over time and may reflect the increasing severity of the opioid epidemic [14,22,23]. Although these studies suggest that analyzing EMS data on naloxone administrations may be a viable approach to monitor opioid overdoses, we are aware of only two studies comparing EMS naloxone administrations before and during the COVID-19 pandemic. Glober et al [24] reported proportional increases in EMS runs for opioid overdoses (43%), EMS-administered naloxone for opioid overdoses (61%), and drug overdose deaths (47%) in Marion County (Indiana) as compared to either of two prepandemic periods. Ballesteros et al [25] reported increases in both EMS-administered naloxone for opioid-related overdoses (28%) and opioid overdose deaths (15%) in Michigan from March 1 to September 2020, as compared with the same period in 2019. To our knowledge, no study has examined changes in multiple naloxone administrations during the COVID-19 pandemic.

This study examines changes in naloxone administrations during EMS runs for opioid-related overdoses during the COVID-19 pandemic in Guilford County, the third most populous county in North Carolina (NC). We focused on Guilford County for two reasons. First, the confluence of the opioid crisis and COVID-19 in Guilford County is evident. The number of opioid overdose deaths increased in the county during the study period (47 in 2015, 71 in 2016, 98 in 2017, 96 in 2018, and 108 in 2019) [1,26]. Likewise, COVID-19 deaths in the county increased and, by the end of the study period, recorded a fatality rate (35.3 per 100,000) resembling the rate for NC (34.9 per 100,000) [27]. Second, one of the study investigators secured an ongoing data use agreement with the Guilford County EMS department to regularly share EMS data. For these reasons, we compared opioid overdose–related EMS runs, naloxone administrations, and multiple naloxone administrations before and during the COVID-19 pandemic in Guilford County.

## Methods

### Data

This study used data from the Guilford County, NC EMS department on opioid overdose–related EMS runs from January 1, 2014, to September 30, 2020. These dates correspond respectively to the earliest and latest data available to us prior to manuscript submission. Opioid overdose–related runs were identified using the fields *primary impression* and *secondary impression*, as indicated by the EMS personnel for the reasons for the encounter. The data set also included an observation for each treatment (eg, naloxone) administered during an opioid overdose–related EMS run. Because the data set was deidentified, we used a combination of the incident date, patient’s birth date, and patient’s gender to generate a quasi-unique run identifier to identify runs that included multiple naloxone administrations. We then grouped the data set to the unit of analysis of opioid overdose–related EMS runs and calculated weekly counts by calendar week to allow for comparison over time.

### Outcomes

We studied three weekly count outcomes: (1) opioid overdose–related EMS runs, (2) naloxone administrations during opioid overdose–related EMS runs, and (3) multiple naloxone administrations during opioid overdose–related EMS runs. Together, these outcomes permit the analyses of change in the occurrence (1 and 2) and severity (3) of opioid overdoses [14,16-20,22,23].

To measure change in these outcomes in the 29 weeks following NC’s COVID-19 state of emergency declaration on March 10, 2020 (ie, the COVID-19 period), we calculated the mean of each outcome during the 29-week COVID-19 period, then expressed it as a percent change from the mean of the outcome during a comparison period.

### Period-Over-Period Approach

We evaluated period-over-period change between the COVID-19 period and two other comparison periods. For the first, we compared the COVID-19 period to the 29 calendar weeks immediately preceding the state of emergency declaration (September 1, 2019, to March 9, 2020). For the second, we compared the COVID-19 period to the same 29 calendar weeks of the previous year (March 13 to October 5, 2019). The second comparison accounts for potential seasonality in opioid overdoses [28]. We chose to include the second comparison after time series decomposition showed yearly seasonality in opioid overdose–related EMS runs.

This period-over-period approach is a generalization of the year-over-year growth rate commonly used in finance and business analytics [29]. The period-over-period changes between the COVID-19 period and the comparison periods can be considered the absolute effect size. To test the hypothesis that the COVID-19 period and comparison periods had unequal means, we conducted Welch unequal variances *t* tests for each outcome and comparison period. We treated the means in each 29-week period as independent samples. We used a critical value of 0.05 and two-tailed tests. Although these comparisons depict the magnitude of change of the outcomes during the COVID-19 period, they alone cannot convey how unique such a change is compared to more distal past periods. What may seem like a large increase may not be noteworthy (nor connected to the COVID-19 pandemic) if changes of similar magnitude occurred frequently in the past. Therefore, we used historical data to generate a quasi-control distribution of period-over-period changes to compare to the change observed during the COVID-19 period.

This produced 270 possible comparisons of a 29-week period to the previous 29 weeks, and 246 possible comparisons of a 29-week period to the same 29 weeks of the previous year. We repeated this process for both comparison periods. We then compared the change during the COVID-19 period to the distribution of past period-over-period changes. This provides a measurement of how extreme the change in outcomes during the COVID-19 period was relative to past changes.

### Illustration of Period-Over-Period Approach

**Figure 1:**
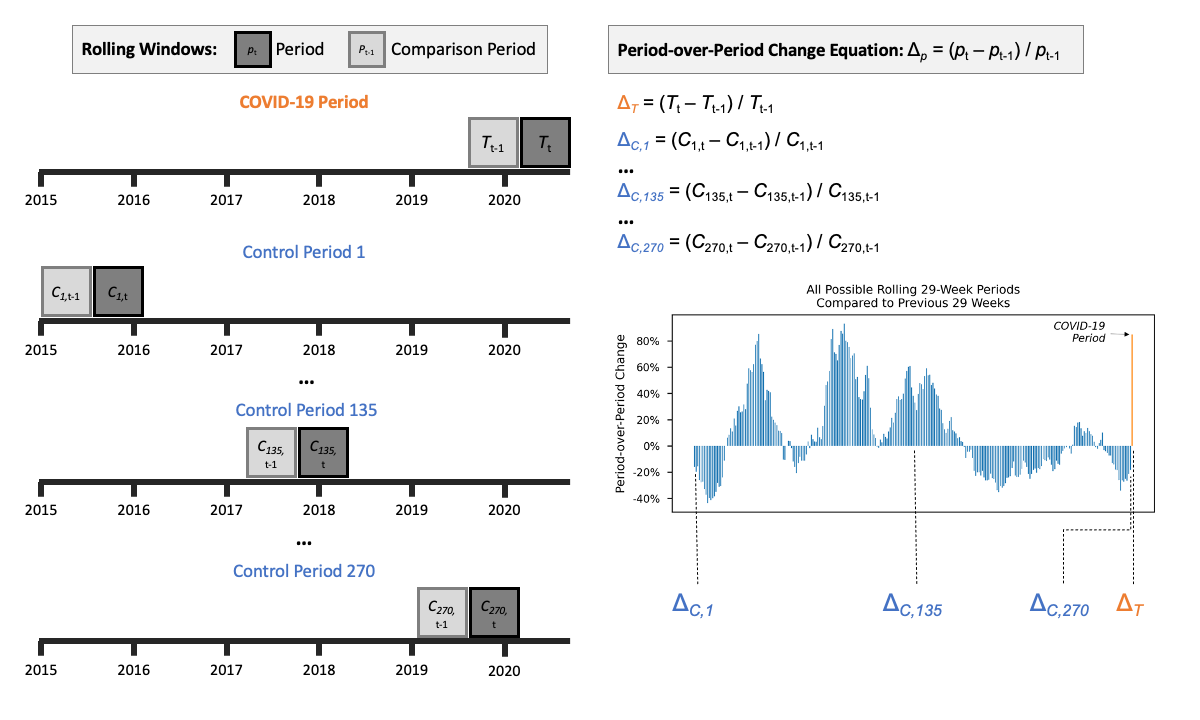
Illustration of period-over-period change method using previous 29 weeks as comparison period from January 1, 2015, to September 30, 2020.

Figure 1 depicts the process used to calculate these period-over-period changes for the COVID-19 period (shown in orange) and the comparison periods (shown in blue). In the top timeline, the period-over-period change for the COVID-19 period is calculated by comparing the outcome from March 10 to September 30, 2020, to the outcome during the previous 29 weeks. The next three timelines depict how the quasi-control distribution was generated for more distal periods. In the second timeline (control period 1), the first control observation was calculated by comparing the outcome from January 1 to July 18, 2015, with the outcome from July 19, 2015, to January 30, 2016. The ellipses indicate that, from this starting point to the beginning of the COVID-19 state of emergency declaration, a control observation was calculated for each calendar week.

### Statistical Analyses

The equations in Figure 1 show how period-over-period change was calculated for each period. This analytic approach is similar in concept to the common difference in difference (DID) quasi-experimental research design [30]. In DID terms, we treated each possible 29-week period as a unit *p* that is observed twice (*t*, *t*-1). For each period, *p*_t_ is the mean of the outcome during the primary period and *p*_t-1_ is the mean of the outcome during the comparison period (either the previous 29 weeks or the same 29 weeks of the previous year). We calculated the percent change in period *p* as Δ*_p_* = (*p*_t_ – *p*_t-1_) / *p*_t-1_. We can consider the COVID-19 period to be the treatment group, which is exposed to the treatment condition (the COVID-19 pandemic) in *t* and to the control condition in *t*-1. The distribution of past period-over-period changes forms the control group, which is exposed to the control condition in both *t* and *t*-1. This results in a control group of n=270 or n=246 (depending on the comparison period used) and a treatment group of n=1.

This treatment group of n=1 precluded the usual regression analysis and statistical inference used with DID study data. Instead, we used two simple heuristics to express how extreme the change in outcomes during the COVID-19 period was relative to past changes. First, we calculated the percentile of the COVID-19 period (Δ) within the distribution of past changes. This allowed us to determine how often period-over-period changes of greater magnitude had occurred in the past. We then plotted period-over-period changes over time and graphically compared the change in outcomes during the COVID-19 period to the recent trend. The plot shows how each period forms one observation in a timeline where period-over-period change is calculated for each calendar week.

**Figure 2:**
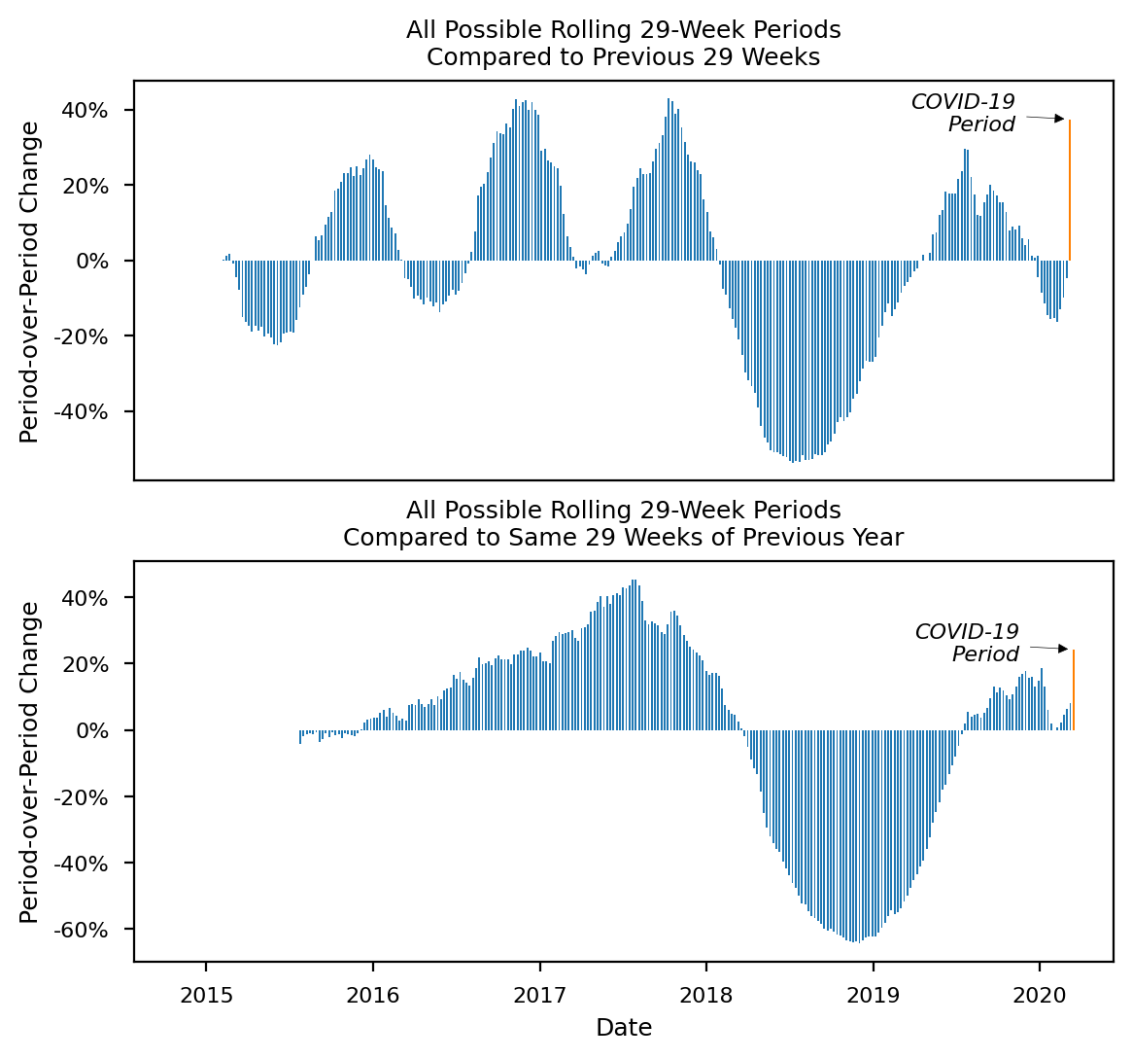
Historical period-over-period change in emergency medical services runs involving opioid overdoses in Guilford County, North Carolina before and after the COVID-19 state of emergency declaration.

**Figure 3:**
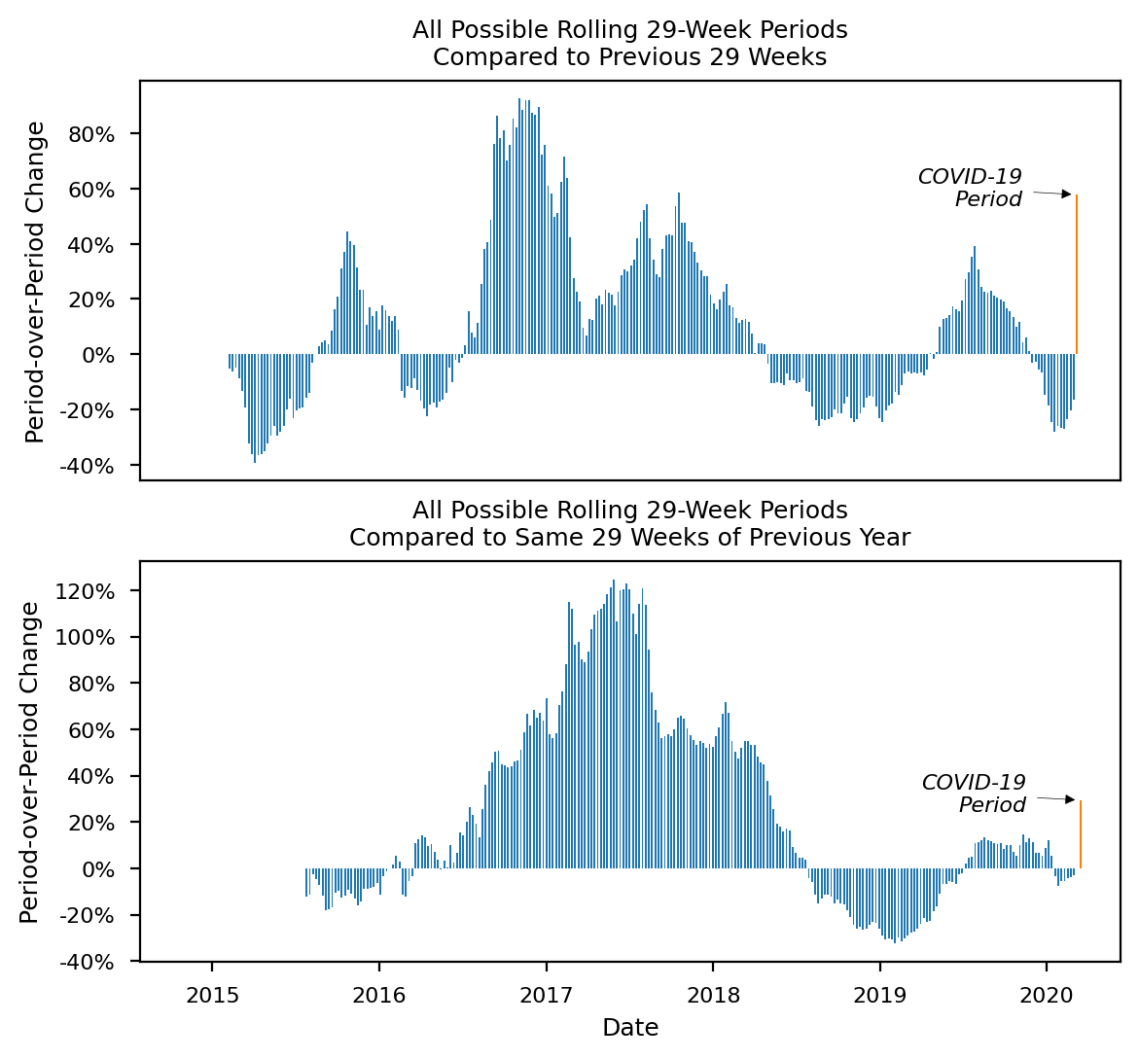
Historical period-over-period change in emergency medical services runs involving naloxone administrations in Guilford County, North Carolina before and after the COVID-19 state of emergency declaration.

**Figure 4:**
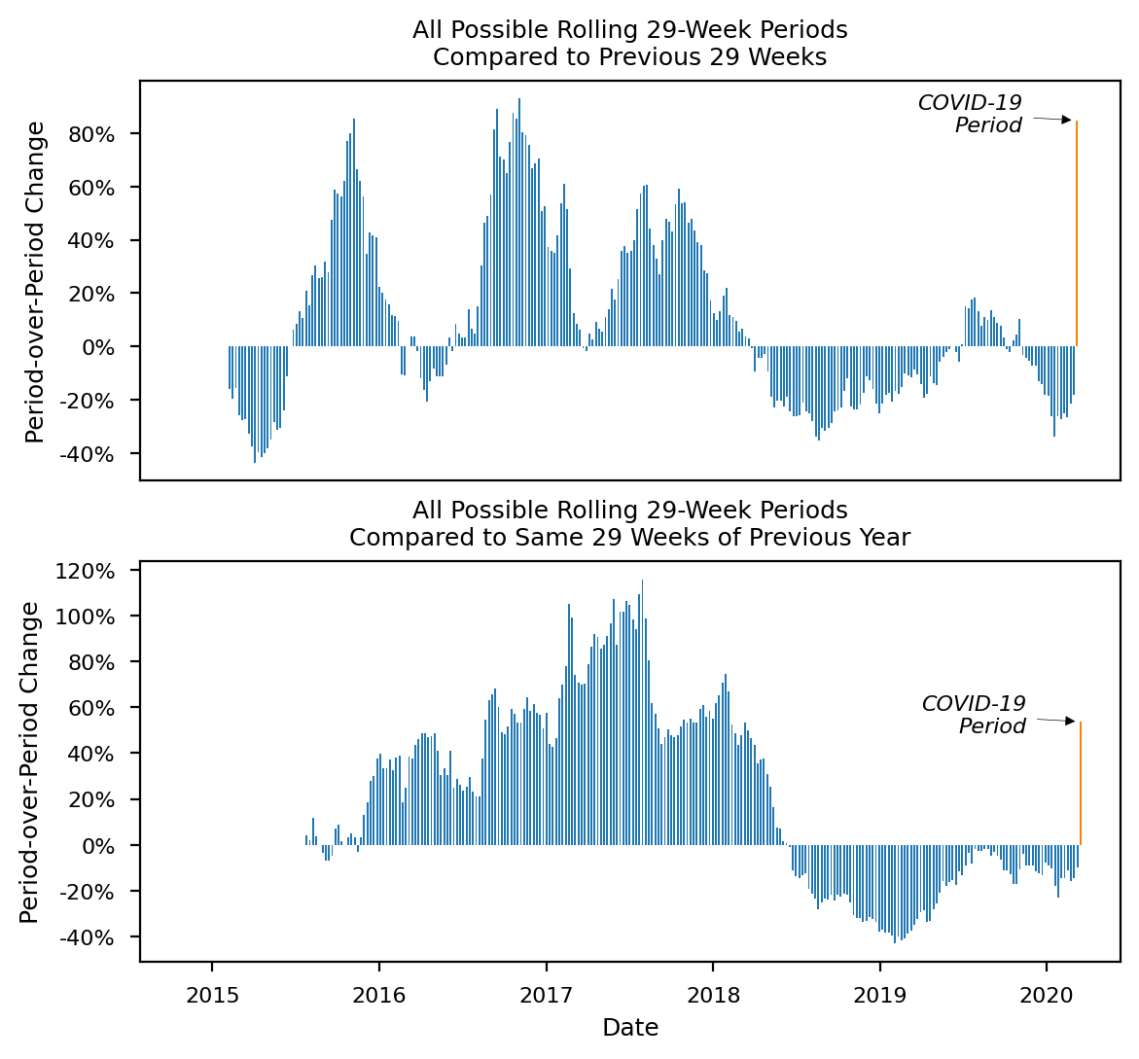
Historical period-over-period change in emergency medical services runs involving multiple naloxone administrations in Guilford County, North Carolina before and after the COVID-19 state of emergency declaration.

Finally, a series of figures further illustrates how the increases in outcomes during the COVID-19 period represent departures from historical trends. For each figure (Figures 2-4), the x-axis represents time from the first possible comparison period (February 8, 2015) since the beginning of the data set (January 1, 2014) to the end of the COVID-19 period (September 30, 2020). The y-axis represents period-over-period change or the percent change in each period relative to its comparison period. Each bar shows a 29-week period’s change from its comparison period, 1 calendar week at a time as previously described. The furthest right bar (in orange) shows the outcome during the COVID-19 period. Period-over-period changes are shown for opioid overdose–related EMS runs (Figure 2), naloxone administrations (Figure 3), and multiple naloxone administrations (Figure 4).

All data analysis was conducted using Python 3.8 (Python Software Foundation) and the Python packages NumPy 1.19.0, SciPy 1.5.0, and pandas 1.0.5.

## Results

All three outcomes increased sharply and significantly during the COVID-19 period (Table 1).

Table 1 shows that, compared to either 29-week comparison period, the mean number of opioid overdose–related EMS runs, naloxone administrations, and multiple naloxone administrations increased during the COVID-19 period. For each outcome, the magnitude of change during the COVID-19 period was greater when compared to the previous 29 weeks than when compared to the same 29 weeks of the previous year. This suggests that seasonality may account for some, but not all, of the increase from the previous 29 weeks.

Further, the increases in outcomes during the COVID-19 period were extreme historically. Table 2 shows, for both comparison periods, the COVID-19 period change expressed as a percentile of the quasi-control distribution of all past period-over-period changes. Across the three outcomes, the increase during the COVID-19 period was greater than 91% of all past 29-week period-over-period changes. When compared to the same 29 weeks of the previous year, the increase for each outcome during the COVID-19 period exceeded at least 63% of all past changes.

**Table 1:** Mean and percent change in EMS runs involving opioid overdoses, naloxone administrations, and multiple naloxone administrations in Guilford County, North Carolina before and after the COVID-19 state of emergency declaration.

Outcomes	COVID-19 period^a^, mean (SD)^b^	Comparison period						
		Previous 29 weeks^c^				Same 29 weeks of previous year^d^		
		Mean (SD)^b^	COVID-19 period change (%)^e,f^	*P* value	Mean (SD)^b^		COVID-19 period change (%)^e^	*P* value
Opioid overdose–related EMS^g^ runs	25.6 (5.6)	18.6 (6.6)	37.4	<.001	20.6 (6.5)		24.3	.003
Naloxone administrations	22.3 (6.2)	14.1 (6.0)	57.8	<.001	17.2 (6.9)		29.7	.006
Multiple naloxone administrations	5.0 (1.9)	2.7 (1.9)	84.8	<.001	3.3 (1.8)		51.5	<.001

^a^March 10 to September 30, 2020.

^b^Weekly mean and SD of outcome during period.

^c^September 2, 2019, to March 9, 2020.

^d^March 13 to October 5, 2019.

^e^(COVID-19 period mean – comparison period mean) / comparison period mean.

^f^The percentage changes shown may not correspond exactly to the values shown due to rounding.

^g^EMS: emergency medical services.

**Table 2:** Magnitude of period-over-period change in EMS runs involving opioid overdoses, naloxone administrations, and multiple naloxone administrations in Guilford County, North Carolina before the COVID-19 state of emergency declaration.

Comparison period		Percentile (%)^a^
**Opioid overdose–related EMS^b^** **runs**		
	Previous 29 weeks^c^	94.8
	Same 29 weeks of previous year^d^	81.7
**Naloxone administrations**		
	Previous 29 weeks^c^	91.1
	Same 29 weeks of previous year^d^	63.0
**Multiple naloxone administrations**		
	Previous 29 weeks^c^	98.1
	Same 29 weeks of previous year^d^	76.8

^a^Percentile of COVID-19 period-over-period change within quasi-control distribution of all past period-over-period changes.

^b^EMS: emergency medical services.

^c^September 1, 2019, to March 9, 2020.

^d^March 13 to October 5, 2019.

All outcomes showed mostly positive period-over-period changes from 2015 to 2017. An inflection point occurred in mid to late 2017 when naloxone administrations and multiple naloxone administrations started to drop (in July 2017), followed by a drop in opioid overdose–related runs (in November 2017). From 2018 to 2020, the outcomes continued to decrease, and most period-over-period changes were negative. In the COVID-19 period, a sharp increase occurred for all outcomes. The large positive period-over-period changes in the COVID-19 period are thus an abrupt and large departure from the recent trend of mostly negative period-over-period changes. Most notably, multiple naloxone administrations increased by 84.8% when compared to the previous 29 weeks and by 53.7% when compared to the same 29 weeks of 2019.

## Discussion

This study detected increases in the occurrence of EMS runs for opioid-related overdoses, naloxone administrations, and multiple naloxone administrations during EMS runs in the course of the COVID-19 pandemic in Guilford County, NC. The findings confirm and extend research on the convergence of the opioid epidemic and the COVID-19 pandemic. The study replicates previous research [11,24,25] by showing increases in opioid overdose–related EMS runs and EMS-administered naloxone during the COVID-19 pandemic in a large metropolitan county in NC. The sharp increase in multiple naloxone administrations, an indicator of the potential lethality of opioid overdoses [13,21,22], constitutes new evidence linking COVID-19 to an increase in the severity of the overdose crisis. Moreover, the period-over-period analytic approach demonstrates unequivocally that the increases observed for each outcome were historically uncharacteristic of prior periods dating back to 2014 and not explained by seasonality effects. Together, these findings indicate that opioid overdoses have increased in occurrence and severity in Guilford County during COVID-19 to a great extent, thereby contributing to growing empirical support for the hypothesis that the COVID-19 pandemic exacerbates the opioid epidemic [31,32].

To help interpret the findings reported here and elsewhere, we refer to a host-agent-vector-environment model to synthesize the multifactorial evolution of the opioid overdose crisis in the United States [33]. Based upon a modification of the epidemiological triangle model of disease for chronic health conditions [34-36], the model posits that the risk of opioid overdose results from an interaction of risk factors associated with the *host* (ie, individual-level factors such as addiction susceptibility or opioid tolerance), the *agent* (ie, external factors such as heroin or fentanyl), the *vector* (ie, purveyors of licit and illicit drugs), and the environment (ie, contextual external factors such as the economy or geography). With this model in mind, this study offers compelling evidence that the COVID-19 pandemic serves as a potent environmental factor that increases the risk of opioid overdoses, perhaps by augmenting the risk-conferring nature of other factors in the model.

There are a variety of ways by which the environmental strain of the COVID-19 pandemic could interact with host, agent, and vector factors to potentiate opioid overdose risk. Host factors (eg, central nervous system depression) associated with overdose risk may intensify under conditions of social isolation by using opioids alone and without the availability of a bystander to administer naloxone [3]. Indeed, Friedman et al [10] reported increases in EMS runs for opioid overdose cardiac arrests that corresponded with decreases in mobility, an indicator of social isolation. Other host factors (eg, tolerance to opioids) may temporarily decrease due to pandemic-related barriers that limit acquiring and using opioids, only to be supplanted with an escalation of overdose risk when opioids become more available and used at prehiatus dosage [3,37]. Consistent with this notion, Currie et al [38] reported an initial fentanyl-fueled spike in opioid overdose deaths in Ohio approximately 6 weeks following the declaration of a national public health emergency on March 13, 2020. Notably, opioid overdose deaths returned to comparable historical levels within approximately 3 months. Regarding agent factors, it is evident that the COVID-19 pandemic began during the third wave of the opioid epidemic when overdoses were increasingly attributable to the use of heroin and fentanyl [39]. With its estimated 50 times potency of heroin, fentanyl and other synthetic opioids substantially intensify the risk of fatal and nonfatal overdoses [40]. Additionally, polysubstance abuse increases overdose risk [41] and has now been empirically linked to risk of opioid overdoses during COVID-19 [26]. Regarding vector factors, COVID-19 may potentiate supply-side pressures that intensify overdose risk [3,42]. Fentanyl increasingly dominates the illegal drug market [43] and remains highly prevalent in drugs tested during the pandemic [9]. National drug positivity rates increased especially for fentanyl and heroin despite a decrease in overall positivity during the study period [8], suggesting a net increase in lethality due to greater exposure of fentanyl in the drugs that were used.

Regarding specific environmental factors, reduced access to treatment and other services for opioid use disorders likely contributed to overdoses [44]. Opioid use disorder treatment and harm reduction services have been substantially impacted by COVID-19 social distancing regulations due to limits on face-to-face contact [45]. Historically, harm reduction services such as syringe exchange programs, drug consumption rooms, naloxone distribution, and fentanyl test strips have been provided in an in-person capacity. In fact, Schlosser and Harris [46] argue that harm reduction services are predicated on the “physical, social, and emotional intimacies” associated with drug use, conditions that were limited after social distancing regulations were introduced. Bartholomew et al [47] found that 15% of needle and syringe programs across nine states closed during COVID-19, 72% were operating at limited capacity, and 25% eventually pivoted to provide virtual harm reduction services. These trends in closures, reduced hours, and service modifications are reflected across the United States [48] and 25 countries in Europe [49].

Our study has several limitations. The use of a convenience sample of EMS records in a single county limits the generalizability of the findings. Although our finding of increased naloxone administrations during COVID-19 replicates a study conducted in Marion County (Indiana) [24], investigations in other geographical locations are needed. By using only EMS data, the use of naloxone by bystanders or other first respondents (eg, law enforcement) are not included. Furthermore, part of the increase in opioid-related EMS runs during the pandemic may be driven by temporary redirection of other treatment access points, perhaps due to a disincentive to seek care at hospitals during the pandemic. There is also a potential for misclassification of overdose events, as EMS responders may overuse naloxone in some cases. Because of their anonymized nature, EMS data were not linked to death records; doing so would help verify multiple naloxone administration as a proxy for severity. It also remains unclear the extent that the increases in naloxone administrations and multiple naloxone administrations reported here are attributable to the COVID-19 pandemic independent of fentanyl’s effect; research on this is needed.

To our knowledge, this study is the first to report an increase in both occurrence and severity of opioid overdoses during the COVID-19 pandemic. Our study also encourages additional research on the use of EMS data on naloxone administrations and multiple naloxone administrations to monitor opioid overdoses. The timeliness, ubiquity, and specificity to the opioid crisis make EMS data on naloxone administrations especially viable proxy measures of the evolving opioid epidemic, even during public health disasters such as the COVID-19 pandemic. More broadly, our study reiterates the value of data sharing among public health and safety, research, community, and academic organizations for tracking the opioid crisis [5,7,50,51]. The global sharing of COVID-19 data clearly shows that this is possible.

## Abbreviations

CDC: Centers for Disease Control and Prevention
DID: difference in difference
EMS: emergency medical services
NC: North Carolina

## References

[1] National Center for Health Statistics Mortality Data on CDC WONDER. CDC WONDER.

[2] Becker W, Fiellin D (2020). When epidemics collide: coronavirus disease 2019 (COVID-19) and the opioid crisis. Ann Intern Med.

[3] Wakeman SE, Green TC, Rich J (2020). An overdose surge will compound the COVID-19 pandemic if urgent action is not taken. Nat Med.

[4] Ahmad FB, Rossen LM, Sutton P (2021). Provisional drug overdose death counts. Centers for Disease Control and Prevention.

[5] Blanco C, Compton WM, Volkow ND (2020). Opportunities for research on the treatment of substance use disorders in the context of COVID-19. JAMA Psychiatry.

[6] Anwar M, Khoury D, Aldridge AP, Parker SJ, Conway KP (2020). Using Twitter to surveil the opioid epidemic in North Carolina: an exploratory study. JMIR Public Health Surveill.

[7] Zibbell JE, Aldridge AP, Cauchon D, DeFiore-Hyrmer J, Conway KP (2019). Association of law enforcement seizures of heroin, fentanyl, and carfentanil with opioid overdose deaths in Ohio, 2014-2017. JAMA Netw Open.

[8] Niles JK, Gudin J, Radcliff J, Kaufman HW (2021). The opioid epidemic within the COVID-19 pandemic: drug testing in 2020. Popul Health Manag.

[9] Wainwright JJ, Mikre M, Whitley P, Dawson E, Huskey A, Lukowiak A, Giroir BP (2020). Analysis of drug test results before and after the US declaration of a national emergency concerning the COVID-19 outbreak. JAMA.

[10] Friedman J, Beletsky L, Schriger DL (2021). Overdose-related cardiac arrests observed by emergency medical services during the US COVID-19 epidemic. JAMA Psychiatry.

[11] Slavova S, Rock P, Bush HM, Quesinberry D, Walsh SL (2020). Signal of increased opioid overdose during COVID-19 from emergency medical services data. Drug Alcohol Depend.

[12] Lerner E, Newgard C, Mann N (2020). Effect of the coronavirus disease 2019 (COVID-19) pandemic on the U.S. Emergency Medical Services System: a preliminary report. Acad Emerg Med.

[13] Cash RE, Kinsman J, Crowe RP, Rivard MK, Faul M, Panchal AR (2018). Naloxone administration frequency during emergency medical service events - United States, 2012-2016. MMWR Morb Mortal Wkly Rep.

[14] Faul M, Lurie P, Kinsman JM, Dailey MW, Crabaugh C, Sasser SM (2017). Multiple naloxone administrations among emergency medical service providers is increasing. Prehosp Emerg Care.

[15] Rzasa Lynn R, Galinkin JL (2018). Naloxone dosage for opioid reversal: current evidence and clinical implications. Ther Adv Drug Saf.

[16] Faul M, Dailey MW, Sugerman DE, Sasser SM, Levy B, Paulozzi LJ (2015). Disparity in naloxone administration by emergency medical service providers and the burden of drug overdose in US rural communities. Am J Public Health.

[17] Knowlton A, Weir BW, Hazzard F, Olsen Y, McWilliams J, Fields J, Gaasch W (2013). EMS runs for suspected opioid overdose: implications for surveillance and prevention. Prehosp Emerg Care.

[18] Lindstrom HA, Clemency BM, Snyder R, Consiglio JD, May PR, Moscati RM (2015). Prehospital naloxone administration as a public health surveillance tool: a retrospective validation study. Prehosp Disaster Med.

[19] Moore PQ, Weber J, Cina S, Aks S (2017). Syndrome surveillance of fentanyl-laced heroin outbreaks: Utilization of EMS, Medical Examiner and Poison Center databases. Am J Emerg Med.

[20] Ray BR, Lowder EM, Kivisto AJ, Phalen P, Gil H (2018). EMS naloxone administration as non-fatal opioid overdose surveillance: 6-year outcomes in Marion County, Indiana. Addiction.

[21] Lasher L, Rhodes J, Viner-Brown S (2019). Identification and description of non-fatal opioid overdoses using Rhode Island EMS data, 2016-2018. R I Med J (2013).

[22] Klebacher R, Harris M, Ariyaprakai N, Tagore A, Robbins V, Dudley LS, Bauter R, Koneru S, Hill RD, Wasserman E, Shanes A, Merlin MA (2017). Incidence of naloxone redosing in the age of the new opioid epidemic. Prehosp Emerg Care.

[23] Somerville NJ, O'Donnell J, Gladden RM, Zibbell JE, Green TC, Younkin M, Ruiz S, Babakhanlou-Chase H, Chan M, Callis BP, Kuramoto-Crawford J, Nields HM, Walley AY (2017). Characteristics of fentanyl overdose - Massachusetts, 2014-2016. MMWR Morb Mortal Wkly Rep.

[24] Glober N, Mohler G, Huynh P, Arkins T, O'Donnell D, Carter J, Ray B (2020). Impact of COVID-19 pandemic on drug overdoses in Indianapolis. J Urban Health.

[25] Ballesteros A, Roche J, Myers M, Carter P, Cunningham R, Goldstick J (2021). 0058 Changes in suspected overdoses following the start of the COVID-19 pandemic: results from the michigan system for opioid overdose surveillance. Inj Prev.

[26] COVID-19 North Carolina Dashboard. NCDHHS COVID-19 Response.

[27] Precision for COVID.

[28] Dasgupta N (2013). Opioid analgesic prescribing and overdose mortality in North Carolina. Carolina Digital Repository.

[29] Amadeo K (2020). Year-over-year explained with its pros and cons. The Balance.

[30] Wing C, Simon K, Bello-Gomez RA (2018). Designing difference in difference studies: best practices for public health policy research. Annu Rev Public Health.

[31] Kuehn BM (2021). Accelerated overdose deaths linked with COVID-19. JAMA.

[32] Alexander G, Stoller K, Haffajee R, Saloner B (2020). An epidemic in the midst of a pandemic: opioid use disorder and COVID-19. Ann Intern Med.

[33] Compton W, Jones C (2019). Epidemiology of the U.S. opioid crisis: the importance of the vector. Ann N Y Acad Sci.

[34] Giovino GA (2002). Epidemiology of tobacco use in the United States. Oncogene.

[35] Merikangas K, McClair V (2012). Epidemiology of substance use disorders. Hum Genet.

[36] Merikangas KR, Nakamura EF, Kessler RC (2009). Epidemiology of mental disorders in children and adolescents. Dialogues Clin Neurosci.

[37] Binswanger I, Stern M, Deyo R, Heagerty PJ, Cheadle A, Elmore JG, Koepsell TD (2007). Release from prison--a high risk of death for former inmates. N Engl J Med.

[38] Currie JM, Schnell MK, Schwandt H, Zhang J (2021). Trends in drug overdose mortality in Ohio during the first 7 months of the COVID-19 pandemic. JAMA Netw Open.

[39] Dasgupta N, Beletsky L, Ciccarone D (2018). Opioid crisis: no easy fix to its social and economic determinants. Am J Public Health.

[40] O'Donnell J, Gladden RM, Goldberger BA, Mattson CL, Kariisa M (2020). Notes from the field: opioid-involved overdose deaths with fentanyl or fentanyl analogs detected - 28 States and the District of Columbia, July 2016-December 2018. MMWR Morb Mortal Wkly Rep.

[41] Compton W, Valentino R, DuPont R (2021). Polysubstance use in the U.S. opioid crisis. Mol Psychiatry.

[42] Ciccarone D (2019). The triple wave epidemic: supply and demand drivers of the US opioid overdose crisis. Int J Drug Policy.

[43] Althoff KN, Leifheit KM, Park JN, Chandran A, Sherman SG (2020). Opioid-related overdose mortality in the era of fentanyl: monitoring a shifting epidemic by person, place, and time. Drug Alcohol Depend.

[44] (2021). The Global State of Harm Reduction 2020. Harm Reduction International.

[45] Dietze PM, Peacock A (2020). Illicit drug use and harms in Australia in the context of COVID-19 and associated restrictions: anticipated consequences and initial responses. Drug Alcohol Rev.

[46] Schlosser A, Harris S (2020). Care during COVID-19: drug use, harm reduction, and intimacy during a global pandemic. Int J Drug Policy.

[47] Bartholomew TS, Nakamura N, Metsch LR, Tookes HE (2020). Syringe services program (SSP) operational changes during the COVID-19 global outbreak. Int J Drug Policy.

[48] Glick N, Prohaska S, LaKosky P, Juarez AM, Corcorran MA, Des Jarlais DC (2020). The impact of COVID-19 on syringe services programs in the United States. AIDS Behav.

[49] (2020). EMCDDA trendspotter briefing - impact of COVID-19 on drug services and help-seeking in Europe. European Monitoring Centre for Drugs and Drug Addiction.

[50] Alter A, Yeager C (2020). The consequences of COVID-19 on the overdose epidemic: overdoses are increasing. Overdose Detection Mapping Application Program.

[51] (2019). Guilford County Solution to the Opioid Problem.

